# Electronic Conduction in Ti/Poly-TiO_2_/Ti Structures

**DOI:** 10.1038/srep29624

**Published:** 2016-07-12

**Authors:** Faramarz Hossein-Babaei, Navid Alaei-Sheini

**Affiliations:** 1 Electronic Materials Laboratory, Electrical Engineering Department, K. N. Toosi University of Technology, Tehran 16317-14191, Iran

## Abstract

Recent intensive investigations on metal/metal oxide/metal structures have targeted nanometric single grain oxides at high electric fields. Similar research on thicker polycrystalline oxide layers can bridge the results to the prior literature on varistors and may uncover novel ionic/electronic features originating from the conduction mechanisms involving grain boundaries. Here, we investigate electronic conduction in Ti/poly-TiO_2−x_/Ti structures with different oxygen vacancy distributions and describe the observed features based on the motion and rearrangement of the ionized oxygen vacancies (IOVs) on the grain facets rather than the grain interiors. Containing no interface energy barrier, Ti/poly-TiO_2_/Ti devices demonstrate high resistance ohmic conduction at biasing fields below 5 × 10^6^ V.m^−1^; higher fields drive the samples to a distinctly nonlinear and hysteretic low resistance status. The observed threshold is two orders of magnitude smaller than the typical resistance switching fields reported for the nanosized single grain memristors. This is consistent with the smaller activation energies reported for the IOV motion on the rutile facets than its interior. The presented model describes the observed dependence of the threshold field on the relative humidity of the surrounding air based on the lower activation energies reported for the hydroxyl-assisted IOV motion on the rutile facets.

Nanometric device dimensions and operation at high biasing electric fields have made tracing ionic displacements significant in determining the electronic features in nanoelectronic devices. Ion movements can cause structural alterations and render the current vs. voltage (I–V) characteristics of the device hysteretic^1^,^2^. Profound resistance switching, observed in nanostructured metal oxide semiconductors^3^,^4^,^5^,^6^,^7^, originates from the alteration of the conduction mechanism or route caused by the motion and reformatting of the distribution patterns of the present mobile ionic species^8^. The devices operating on such switching effects (whether or not we call them “memristors”^9^,^10^,^11^) are potentially viable memory cells for future nanoelectronic circuits^12^,^13^,^14^. The structures considered are mostly of the M′/MO/M″ type, where MO is a nanometer-sized metal oxide crystallite sandwiched between M′and M″ metal electrodes^5^,^15^. The most widely studied oxide for resistance switching is TiO_2_ connected to noble metal electrodes, such as platinum^16^,^17^,^18^,^19^,^20^, silver^21^,^22^,^23^ or gold^24^,^25^, which usually add to the complexity of the device performance by forming Schottky energy barriers at their junctions with TiO_2_^26^,^27^,^28^, and introduce at least one additional mobile cation to the structure^23^.

The motion, assembling, and relaxation of the mobile ionic species at varying electric field levels form or eliminate different electron conduction routes and result in unpredictable I–V characteristics. While the migration of the ionized oxygen vacancy (IOV) has been established as the most effective cause of resistance variation in these devices^5^,^29^,^30^,^31^,^32^,^33^,^34^, it has been shown that the motions of all cations, including those originating from the electrodes, can determine the main conduction mechanism and promote hysteretic behaviors^3^,^8^,^35^. It appears that a better understanding of the device performance can be achieved in Ti/TiO_2−x_/Ti samples in which candidate mobile species are limited in nature. Moreover, unlike the case of many other transition metals^36^,^37^, no energy barrier is formed at the junction between titanium and TiO_2_^38^,^39^,^40^, making the electronic performance of the device simpler to model.

Mainly in the 70 s and 80 s, resistance switching in metal oxides, particularly in ZnO, was the subject of many fruitful investigations resulting in a large family of electroceramic voltage dependent resistors (varistors) and surge arrestors which still dominate the market^41^,^42^,^43^,^44^,^45^. (Varistors are generally polycrystalline resistors with considerably smaller resistances at higher applied voltages.) However, regardless of their seemingly similar metal oxide-based resistance switching mechanisms, recent research on nanometric crystallites remains isolated from the prior literature concerning millimeter-thick metal oxide disks as they use different methods to different ends. Investigating the electronic features of Ti/poly-TiO_2_/Ti structures with dimensions between nanometric resistive memory devices and milimetric varistors can bridge the gap between these two technologies and advance the state of the art in both fields. For instance, recently recorded observations concerning the mechanism of point defect motion on oxide grain facets^46^,^47^,^48^,^49^ may clarify the obscure ionic/electronic conduction through the oxide grain boundaries in varistors leading to enhanced quality factors in new device designs. Likewise, introducing methods of conduction control in the intergranular phase of wide band gap semiconductors^50^ to the relatively novel subject of nanosized resistive memory devices^3^,^4^,^5^,^6^,^7^ can help initiate the production of surface conduction-based memory devices, rather than bulk conduction-based ones.

Here, we study the I–V characteristics of the Ti/poly-TiO_2−x_/Ti structures made of ~400 nm-thick oxide layers with different cross-sectional oxygen vacancy distribution profiles and describe the results based on two common conduction mechanisms prevailing respectively in the low and high biasing electric fields. Using the latest advances in observing and understanding the oxygen vacancy motion on rutile surfaces^46^,^47^, we show that the resistance switching mechanism in the polycrystalline samples is restricted to the intergranular regions where the field intensity required for IOV motion is considerably less than that within the grains. It is confirmed that the observed resistance switching in our samples is distinctly different from the similar effect taking place in a single grain resistive memory cell.

## Results and Discussion

**Figure 1:**
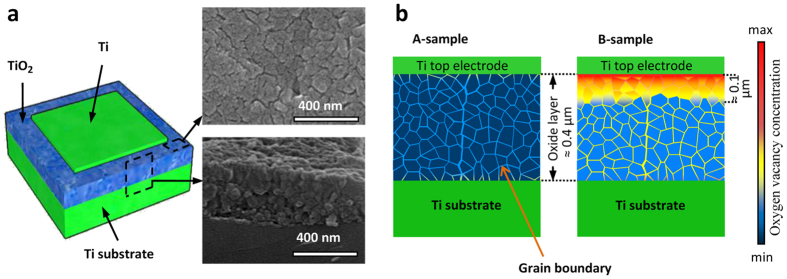
The structure of the samples. (**a**) The schematic diagram of the device structure; insets are the plan and cross-sectional micrographs of the TiO_2_ layer and (**b**) the schematic presentations of the different vacancy distributions in the A-samples and the B-samples.

Polycrystalline rutile layers, dominantly composed of {110}-faceted grains, are grown by the thermal oxidation of pure titanium chips. The fabrication of Ti/poly-TiO_2−x_/Ti samples (Fig. 1a) is completed by selectively depositing titanium thin film on the oxide surface. Representing the two different construction methods (see Methods) are two sample groups, A- and B-samples, which differ in their thermal annealing processes and IOV concentrations, as schematically shown in Fig. 1b.

**Figure 2:**
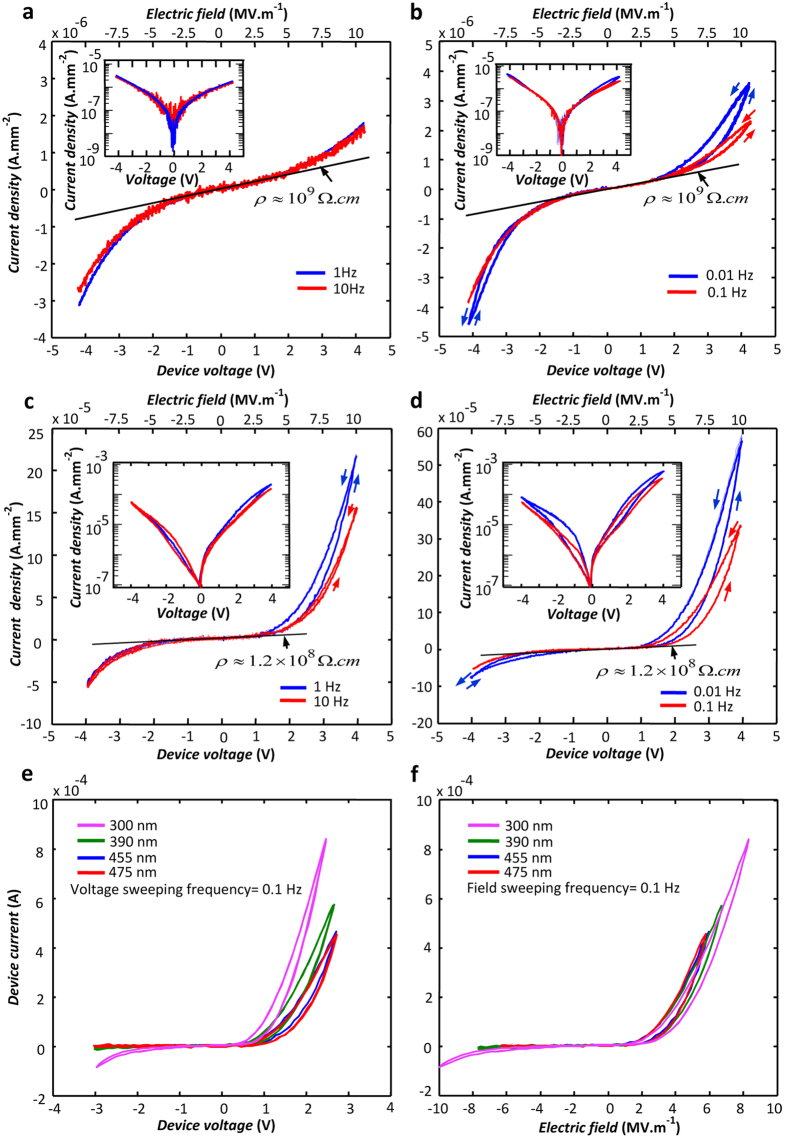
The experimental current density vs. voltage diagrams of different samples. The diagrams are plotted at the stated voltage sweeping frequencies for an A-sample (**a**,**b**) and for a B-sample (**c**,**d**); both samples are made of 400 nm thick oxide layers; the respective semi-logarithmic plots of the diagrams are presented as insets. The diagrams obtained for four B-samples with the stated thicknesses (**e**) match when presented in the current density vs. electric field form (**f**). All measurements are carried out in clean air with 23% relative humidity at room temperature.

The I–V characteristics obtained for an A-sample in the biasing voltage range of +/− 4 V and voltage sweeping frequencies of 1 and 10 Hz are shown in Fig. 2a in both linear and logarithmic scales. The applied voltage is considered positive when the thin film Ti electrode is positively biased with respect to the Ti substrate. The applied electric field, E, is calculated by dividing the applied voltage by the oxide thickness. The characteristics presented in Fig. 2a are consistent with the symmetric structure of the device and zero junction energy barriers at both Ti/TiO_2_ interfaces^38^,^39^,^40^. A minor departure from symmetry occurs at 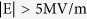 due to the slightly higher IOV concentration in the oxide layer adjacent to the substrate (Fig. 1b). The dynamic resistivity of the oxide layer, determined from the slope of the linear scale I–V in Fig. 2a, is almost independent from the applied field in the +/−2.0 MV/m range; this resistivity, 1.0 GΩ.cm, defines the high resistance state (HRS) of the A-samples at ca. 2.2 MΩ. The I–V diagrams produced for the same sample at lower voltage sweeping frequencies are presented in Fig. 2b. These diagrams lead to the same HRS values at low applied field levels, but indicate hysteretic behavior and higher conduction levels at 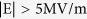 defining the low resistance state (LRS) of the device. The LRS measured at 10 MV/m and 0.01 Hz, is 0.5 MΩ. The device current almost doubles at slow voltage sweeping rates (compare **a** and **b** in Fig. 2), indicating the appearance of a different conduction route related to mobile ions.

The I–V diagrams shown in Fig. 2c,d are plotted for a B-sample at conditions similar to Fig. 2a,b, respectively. At the low applied field range, B-samples demonstrate I–V features similar to those of A-samples, but their HRS, 0.3 MΩ, is an order of magnitude less than that of A-samples. In this mode of operation, the sample resistance is mainly determined by the lowest carrier concentration region in the oxide cross-section, and, hence, the smaller HRS in B-samples is attributed to the higher overall conduction band electron concentrations resulting from their higher IOV population (Fig. 1b). The space-charge-limited electron conduction^51^ in the high resistance oxide accounts for the observed parabolic I–V variations in the low field regions of both sample types. However, B-samples respond differently to positive and negative biasing voltages: responses to negative applied fields resemble those of the A-samples, but upon positive biasing, and particularly at lower sweeping frequencies, the device permits currents two orders of magnitude larger (Fig. 2d). This striking asymmetry in the I–V profiles is attributed to the asymmetric distribution of the IOVs in the oxide cross-section of B-samples schematically presented in Fig. 1b. An hour of thermal annealing at 300 °C in air removes this asymmetry by compensating for the extra oxygen vacancies and converts a B-sample to an A-sample.

The semilogarithmic I–V diagrams, presented as insets in Fig. 2a–d, can reveal if any of the observed I–V asymmetries were due to unexpected energy barrier formed at a metal/oxide or oxide/oxide junction. After a number of attempts, we failed to describe the forward bias segments of the insets in Fig. 2c,d based on the thermionic-emission theory^51^ as the fittings resulted in unacceptably large (>20) ‘ideality factors’^51^ (see Supplementary, Section 3). Lack of junction energy barrier throughout the device cross-sections can also be deduced from Fig. 2e,f which respectively present the I–V and I-E diagrams for four B-samples with different thicknesses. While the I–V plots shown in Fig. 2e are different, their respective I-E diagrams match together, setting them apart from energy barrier devices.

The I–V diagrams shown in Fig. 2 indicate a different conduction mechanism which operates at high biasing fields and switches the electrical resistance to a lower level. On-setting fields are almost the same (ca. 2.5 MV/m) for both negative and positive biases in all A- and B-samples, but the device resistance decreases by two orders of magnitude only in the positively biased B-samples. The high field features of all samples are basically attributed to the motion and filament formation by the IOVs, which reportedly causes the resistance switching in rutile nanocrystals^5^,^6^. Driven by high fields, the IOVs move within the oxide for forming filaments parallel to the field direction^24^,^30^,^31^ and create easy paths for the electrons.

However, according to prior reports, the field intensity required for IOV motion along the [110] direction in rutile is ca. 1.0 GV/m^29^, close to three orders of magnitude larger than field intensity that switches the resistance status in our samples. This discrepancy is explained by considering the IOV motion on the {110} facets of the grains rather than in the grain interior. The TiO_2_ layers examined in this work are polycrystalline with average grain size of 50 nm (Fig. 1a), and, hence, the 400 nm distance between the lower and upper titanium electrodes is covered with a fine web of grain boundaries. In our rutile layers, the dominant grain facets and, hence, the majority of grain boundaries are of {110} indices (see Supplementary, Section 1), which is reportedly the case for the rutile layers produced by the thermal oxidation of titanium at different conditions, as well^52^. The activation energy of oxygen vacancy motion (∆U_a_) in rutile crystal in <110> directions is 1.1 eV^29^,^53^, and a detectable field-assisted migration has been theoretically proven to require electric fields larger than 1.0 GV/m at room temperature^29^. In comparison, ∆U_a_ obtained for the motion of an oxygen vacancy on a (110) facet of rutile, when assisted by the surface adsorbed O_2_ or H_2_O molecules, has been proven analytically and verified experimentally to be 0.3 eV and 0.2–0.5 eV, respectively^48^,^49^.

**Figure 3:**
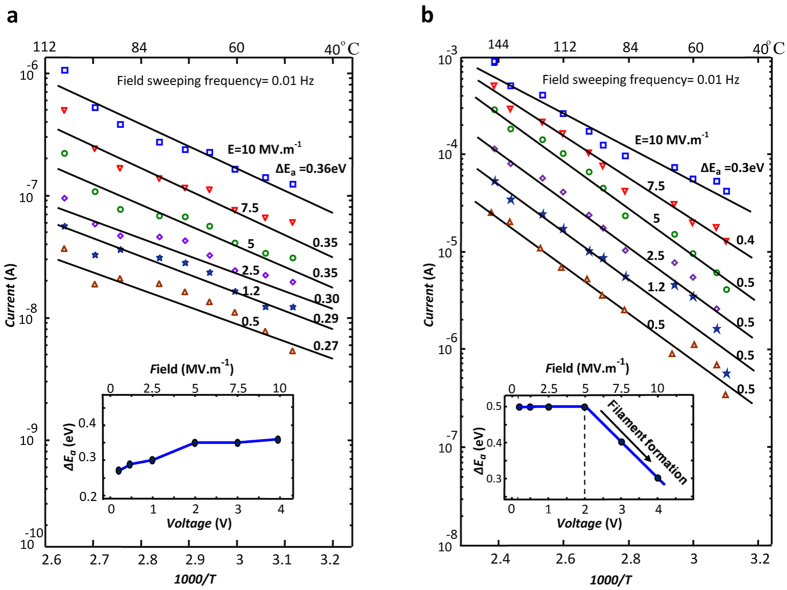
The Arrhenius diagrams of the electric conduction. (**a**) In an A-sample and (**b**) in a B-sample; measurements are carried out at the stated applied fields in clean air with 23% relative humidity. The inset gives the variations of the obtained activation energies with respect to the biasing field.

The activation energy of the electronic conduction (ΔE_a_) (should not be mistaken with the activation energy of IOV motion (∆U_a_)) in both sample categories is investigated by plotting the Arrhenius diagrams of conduction at different biasing fields. The diagrams obtained for A- and B-samples are given in Fig. 3a,b, respectively. As demonstrated in these figures, the estimated ΔE_a_ values in both sample types vary with the applied electric field. The resulted ΔE_a_ vs. applied field plots are given as insets in Fig. 3a,b. The current levels recorded for A-samples (Fig. 3a) are 10^3^ times smaller than those in B-samples (Fig. 3b), allowing electrons to select the easiest path available. There is no significant IOV migration in A-samples, and higher applied fields force the electrons to the non-optimum paths with higher activation energies causing the increase in the overall activation energy deduced from the Arrhenius diagrams (see the inset in Fig. 3a). In B-samples, on the other hand, according to the inset in Fig. 3b, ΔE_a_ of conduction drops from 0.5 eV to 0.3 eV when the electric field is increased from 0.5 MV/m to 10 MV/m. This is attributed to the rearrangement of the IOVs at biasing fields above 5 MV/m, which create easier conduction routes for electrons. Biasing fields insufficient to cause IOV motion hardly affect ΔE_a_ which remains independent from the external field at 0.5 eV. Assuming electron hopping between IOVs to be the foremost conduction mechanism^54^, the observed reduction in ΔE_a_ is consistent with the IOV motion for filament formation. This conduction mechanism is complex, involving electron hopping between traps whose average distance varies with the progress of the filament formation process. A quantitative description of the forward segment in the I–V diagram of a B-sample, hence, requires development of a novel model.

It is arguable that, as the field grows, the motion and reformatting of the IOVs on the grain boundaries parallel with {110} facets establish easy conduction routes for the electrons. To prove that the applied fields in 10 MV/m range are enough for IOV motion on a (110) surface, we calculate the speed of field-assisted migration for the IOVs with two different ∆U_a_ of motion. The calculations are based on the approximate relationship proposed decades ago^55^ to connect the motion of the ionic species in a solid to the ΔU_a_ of their displacement mechanism and the applied external field. This relationship has been modified and used for IOV motion calculations in rutile in the following form^29^:


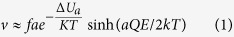


in which *f* is the attempt frequency (10^13^ Hz as given in reference^29^), *a* is the atomic distance in the direction of motion, Δ*U*_*a*_ is the activation energy of motion, and *Q* is the ionic charge.

**Figure 4:**
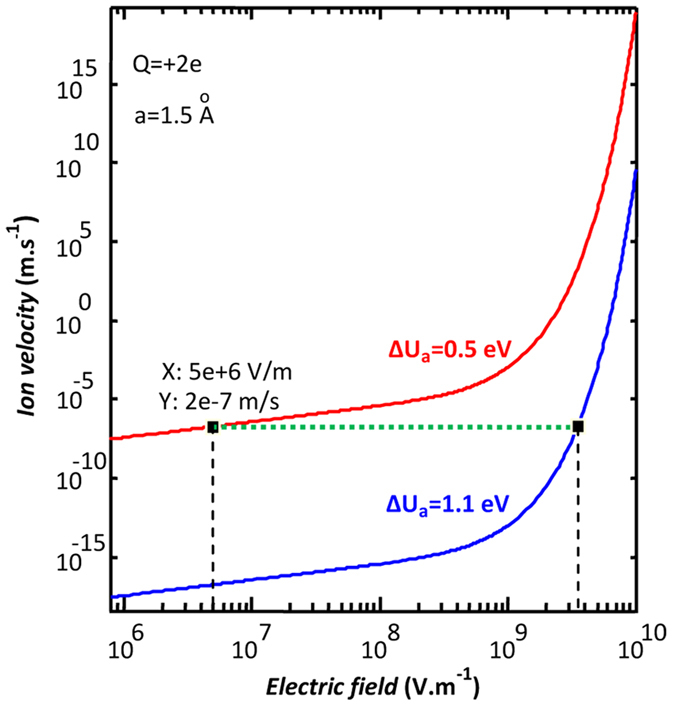
The drift velocities of the doubly ionized oxygen vacancy in/on a rutile grain. Calculations are carried out for the IOV motion in the [110] direction inside (blue) and on a (110) surface (red) of a single crystalline grain, the results are plotted vs. the applied field. The curves intersect the 2 × 10^−7^ m/s tie line (green) at field intensities almost 3 orders of magnitude apart. These comparative calculations are based on the theoretical relationship presented in ref. 29.

The results given in Fig. 4 indicate that the external field intensity required to reach the same speed (see the tie line in Fig. 4) differs by about 3 orders of magnitude among ionic species with respective motion activation energies of 0.5 and 1.1 eV. These comparative calculations explain the observed difference in the onset fields of the single-grain and poly-crystalline Ti/rutile/Ti devices. Figure 4 also shows that the curves obtained for two different activation energies have very different slopes at their points of IOV motion initiation. This translates into a controllable change of resistance with field intensity in the polycrystalline samples compared to the sudden and hardly containable change of status in a single grain device. This is verified by observing the controllability of the operating point all over the I–V diagrams of our samples.

**Figure 5:**
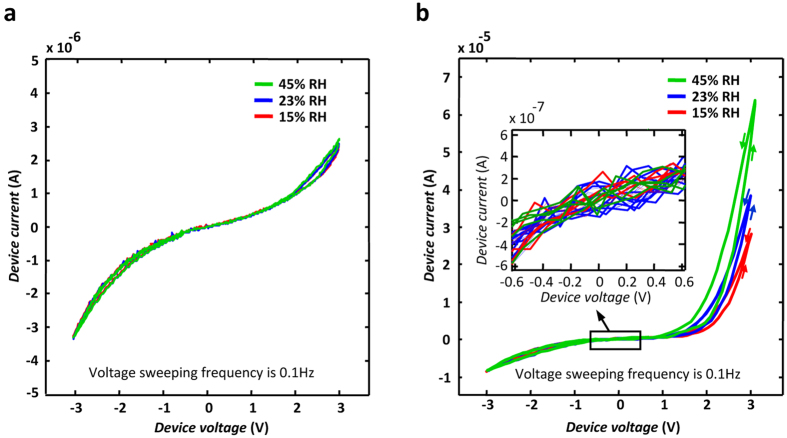
The effect of relative humidity change on the current vs. voltage characteristics of the samples. The diagrams are recorded for an A-sample (**a**) and a B-sample (**b**) in clean air at the stated relative humidity levels at room temperature. The inset in (**b**) magnifies the low voltage region of the diagram to clarify its independence from the relative humidity level.

The function of the adsorbed water monomers in facilitating the IOV motion at the grain boundaries is studied by observing the effect of atmospheric humidity change on the I–V diagrams of the A- and B-samples. The I–V characteristics of the samples are plotted in air at different relative humidity (RH) levels. The results are presented in Fig. 5a,b. While the conduction in the A-sample remains almost unaffected by the humidity change, that in the B-sample increases with the RH level in the surrounding atmosphere. Figure 5b depicts considerable reduction in the switching field with respect to the increased humidity. The observed effect is consistent with the low activation energy reported for the H_2_O molecule-assisted oxygen vacancy motion on the rutile {110} surfaces^46^,^49^. Considering the dense microstructure of the titanium oxide layers grown by thermal oxidation^56^ as well as the lack of humidity-caused change in the HRS of the device (see the inset in Fig. 5b), the effect of humidity is a novel observation distinct from the surface conduction resulting from adsorbed water layers in the porous dielectric materials^57^.

**Figure 6:**
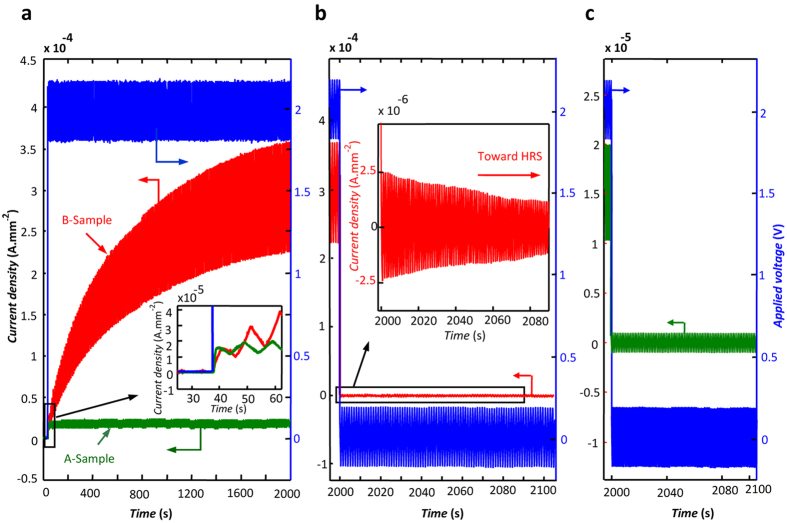
The transient responses of the samples to the sudden changes in the biasing voltage. (**a**) The variations of current in an A-sample (green) and a B-sample (red) in response to a step function biasing voltage (blue), the inset magnifies the responses just after field application. The small AC voltage added to the step function and the recorded AC currents are utilized for dynamic resistance calculations. (**b)** The AC current decay in a B-sample (red) after the sudden removal of the DC bias (blue); although the AC bias has been kept intact, the AC current drops 30 times in less than 10^−3^ s after DC field removal; the inset depicts the gradual diminishing of the residue AC current as the device acquires its HRS. (**c**) The same as (**b**) carried out for an A-sample.

The formation and dissolution mechanisms of IOV filaments are probed by the application of a small AC voltage (V_rms_ = 0.1 V) to the device operating at different DC biasing conditions. The current variations when 2 V step voltages are applied to A- and B-samples are presented in Fig. 6. In A-samples, the current density rises in less than a second to a stable level at 1.5 × 10^−5^ A/mm^2^. In B-samples, however, after a similar fast rise (see the inset in Fig. 6a), the current density increases continuously with time for ~10^3^ s to reach a saturation level at ~20 times above its primary fast rise. Both of these observations are consistent with the filament formation mechanism described above. In both samples, the conduction initiates based on the IOV population present in the grain boundary region, which acquire their minimum resistance format in a second. This is almost the end of the story for A-samples wherein the current increases slightly with time because of the current path enhancement by the IOVs joining the path from the neighboring areas. In B-samples, however, the field-assisted migration of IOVs from the vacancy-rich anode region broadens the filament and gradually enhances the conduction path.

Upon the DC field removal, the dissolution of the IOV filaments occurs immediately (in less than 10^−3^ s) in both A- and B-samples, as shown in Fig. 6b,c. In these experiments, the AC field is kept intact to check the electron conduction mechanism in the samples after the DC field drops from 2 V to zero. The A-sample regains its HRS level immediately. However, in the case of a B-sample, after this major current drop, a residue current remains; as shown in the Fig. 6b inset, regaining the HRS level requires time. The filament dissolution is linked to the thermal diffusion of the IOVs assisted by the repelling coulombic forces between them, which destabilize the filament and disrupt the continuity of the existing electron conducting channel at the absence of the external field. Further experimental results regarding conductive filament formation are provided (see Supplementary, Section 2).

Indeed, the direct observation and concentration estimation of OVs at the cross-section of a 400 nm-thick polycrystalline oxide layer is difficult, if not impossible. However, considering the combination of the utilized sample fabrication methods and the above given electronic characteristics for the B-samples, the asymmetric distribution of the OV and, hence, the IOV in the oxide cross-section is the only logical scenario. The relationship between the IOV and OV concentrations at a point within the oxide follows from the Fermi-Dirac distribution. Thus, assuming a double-ionization energy of 0.48 eV^58^ for OVs in rutile, the IOV concentration at room temperature is smaller than the OV concentration approximately by a factor of 10^−8^. The OV concentration is exponentially related to both the elevated temperature used for oxidation and the rate of cooling upon quenching to room temperature; a rise in the OV concentration of a rutile single crystal has been detected by X-ray photoelectron spectroscopy after a brief heat treatment at 1037 °C^59^. In the case of A-samples, the grown oxide layer cools down to room temperature gradually overnight in the switched off furnace and the rate of cooling is almost the same throughout the oxide cross-section, while B-samples are quenched fast from 650 °C. The faster temperature drop at the outer surface of the TiO_2_ layer leads to a higher OV concentration, while the temperature drop at the oxide’s interface with the substrate is milder due to the substrate’s thermal capacity. A brief reheating of a B-sample (prior to electrode deposition) and allowing it to cool down gradually in the switched off furnace removes the asymmetric OV distribution and converts it to an A-sample. The reverse conversion is also possible: a few minutes of infrared surface heating in vacuum converts an A-sample to a B-sample.

**Figure 7:**
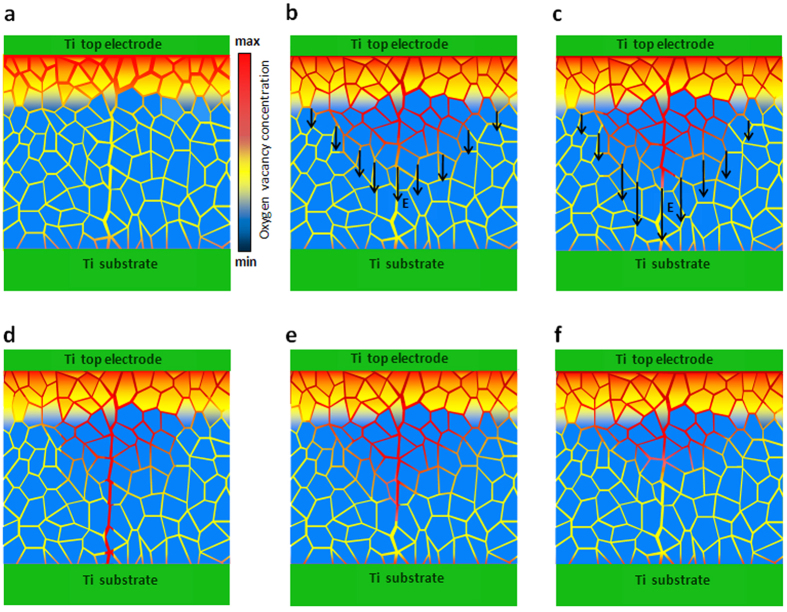
The schematic presentation of the model used for describing the I–V diagram of B-samples. (**a**) The biasing field applied is insufficient to cause IOV motion; the electronic conduction takes place via both grains and grain boundaries; the device is at its HRS. (**b**) At higher biasing fields, IOVs adjacent to the anode move in the field direction via grainboundaries to create conductive filaments; the field intensity is still 100 times smaller than that required for IOV motion within the grains. (**c**) The IOV filament is growing via grain boundaries and the resistance of the sample is still determined by the thinning oxide region with normal grain boundaries. (**d**) The IOV filament connecting the anode to the cathode provides an easy route for the electrons; high current levels through the filament decrease the field intensity in the oxide layer preventing more IOV migration; the device is at its LRS. (**e**) The external field is removed and the repelling coulombic forces have ruptured the filament and increased the device resistance by almost two orders of magnitude; the device is back in its HRS. (**f**) The remaining filament segment is dissolved by thermal diffusion; applying a negative bias would bring the device back to its original status (**a**).

The model schematically presented in Fig. 7a–f describes all observations detailed above. At each cycle of the applied field, the IOV motion at the grain boundaries occurs once the field intensity reaches ca. 2.5 × 10^6^ V/m. At higher field levels, the mobilized IOVs move along grain boundaries to form filaments in directions close to the applied field. The IOV population within the grains does not take part in this process as the applied field is much smaller than that required for their mobilization. The driving force for filament formation comes from the reduced field energy within the oxide layer. The electronic conduction through the formed filament reduces the sample resistance. This field reduction stops further IOV filament development, but filament dissolution occurs when the external field is below 10^6^ V/m. The driving force for filament dissolution originates from the thermal diffusion of the IOVs assisted by the repelling coulombic forces. The hysteretic behavior of the device at high fields is attributed to the two different driving forces acting on the formation and dissolution stages of the IOV filaments. The strong frequency dependence and slowness of the observed phenomena are interpreted based on the fact that both the forming and dissolving of the IOV filaments involve the motion of oxygen ions. The profound asymmetry of the I–V characteristic in B-samples (Fig. 2d) is attributed to the uneven IOV distribution within the oxide layer. (This asymmetric I–V is distinctly different from the I–V asymmetry observed in Schottky type metal/semiconductor junctions^26^,^28^,^51^). In all biasing conditions, the IOVs close to the positively biased electrode play a more constructive role in the filament formation. They are driven away from the positively biased electrode towards the interior of the oxide layer where they are most effective in filament formation. The IOVs adjacent to the cathode, on the other hand, get attracted to the electrode and are less effective in the process. The color coding used in Fig. 7 suggests larger IOV concentration in the grain boundaries than the grains, but the background literature is not clear on this point; opposite IOV concentration variations from inside to the surface of a grain has been reported for ZnO^43^ and SrTiO_3_^60^, and those of TiO_2_ has not yet been experimentally clarified. The model presented here would remain untouched by the direction of those variations.

In summary, we have considered the importance of electronic conduction via the surface IOVs of rutile grains in the operation of memristive devices and demonstrated the vivid differences between the IOV filament formation on the grain surface and in the grain interior. It is shown that the I–V characteristics in Ti/poly-TiO_2_/Ti devices are mainly determined by the surface motion of the IOVs for filament formation and filament dissolution, which have different driving forces and temporal rates. It is clarified that the I–V profile in a Ti/poly-TiO_2−x_/Ti device depends on the prevailing oxygen vacancy distributions profile at the oxide cross-section, which can be altered by the deliberated thermal processing of the oxide layer. Based on the presented conduction model, the dependence of the I–V characteristics of these devices on the relative humidity of the surrounding atmosphere was predicted and experimentally verified.

## Methods

### Fabrication

**Figure 8:**
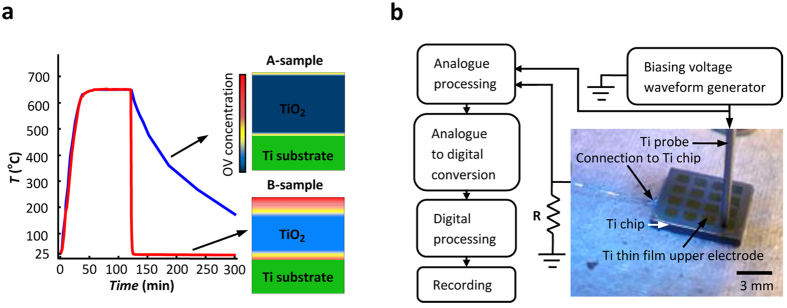
The fabrication and electric characterization methods. (**a**) The temperature vs. time profiles used for the oxidation and quenching of the A-samples (blue) and B-samples (red); the insets schematically demonstrate the cross-sectional oxygen vacancy distribution in the grown oxide layers. (**b**) The photograph of the titanium chip accommodating 16 Ti/TiO_2_/Ti samples positioned on the workbench with the block diagram of the experimental setup used for the I–V recordings.

Titanium oxide layers are grown on 10 mm × 10 mm × 1 mm Ti chips by thermal oxidation at 650 °C for 60 minutes in air. The samples are either cooled over night in the closed chamber of a switched-off furnace (A-samples) or air quenched from the soaking temperature on a corrugated silica-based refractory (B-samples). The temperature vs. time profiles used for the oxidation and quenching of both sample types are given in Fig. 8a. Due to the heat capacity of the Ti substrate and the refractory underneath, the cooling process for the B-samples is profoundly asymmetric and the oxide layer cools faster at the top than the bottom. The thickness of the grown oxide layers is in the 350 nm to 550 nm range. According to the obtained XRD patterns (see Supplementary, Section 1), the grown oxide layers are of rutile phase dominantly consisting of {110}-faceted grains; those grown at 500 °C are mixtures of rutile and anatase^26^. The micrographs given in Fig. 1 reveal a polycrystalline structure with average grain size of 50 nm for the grown oxide layer.

A B-sample can be converted to an A-sample by an hour of thermal annealing at 300 °C in air. Also, due to room temperature annealing, the intense asymmetry of the I–V diagram of a B-sample fades with its holding time in air; the decay time constant is around a week. The Ti/poly-TiO_2_/Ti devices are completed by depositing titanium thin film on the selected areas of the grown oxide layers via the thermal evaporation of the metal in vacuum. Each chip accommodates 16 samples as shown in Fig. 8b.

### Test

The titanium chip acts as the backside electrode and is connected to the measurement circuitry using conductive paste. The top Ti electrode is connected to the measurement system via a pressure-connected titanium probe which can move from one device to another using a manually controlled x-y-z micromanipulator. This method of connection prevents oxide layer contamination with foreign metallic species. All samples are briefly annealed at 60 °C in air and pretested by applying 300 mV AC to the sample connected in series with a 100 Ω resistor; samples allowing currents above 1 mA are rejected. The experimental setup is depicted in Fig. 8b.

Each Ti/poly-TiO_2_/Ti sample is basically a capacitor. The average device capacitance, measured by applying 300 mV AC voltage at 100 Hz, is 2.3 and 3.0 nF.mm^−2^ for the A- and B-samples, respectively. Considering the devices’ geometry and physical dimensions, calculations based on A-samples lead to an average dielectric constant of 130. Placing this value in a similar relationship for a B-sample determines the thickness of the OV-rich region to constitute ~25% of the oxide layer (see Fig. 1b). At a current measurement level of 10^−7^ A, the current contributions of the parallel capacitors are considerable at 10 Hz, which are calculated and deducted from the measured device currents. All measurements are carried out in a Faraday cage.

## Additional Information

**How to cite this article**: Hossein-Babaei, F. and Alaei-Sheini, N. Electronic Conduction in Ti/Poly-TiO_2_/Ti Structures. *Sci. Rep.* **6**, 29624; doi: 10.1038/srep29624 (2016).

## Supplementary Material

Supplementary Information
