# Supplementary material for: Electronic Conduction in Ti/Poly-TiO2/Ti Structures
Source: Sci Rep. 2016 Jul 12;6:29624. doi: 10.1038/srep29624 (PMC4940738; doi:10.1038/srep29624)
Supplement: Supplementary Information [file srep29624-s1.pdf]

Supplementary Information

# Electronic Conduction in Ti/Poly-$TiO_2$/Ti Structures

**Faramarz Hossein-Babaei[1,*] and Navid Alaei-Sheini[1]**

[1]Electronic Materials Laboratory, Electrical Engineering Department, K. N. Toosi University of Technology, Tehran 16317-14191, Iran
fhbabaei@kntu.ac.ir

## 1. Crystal structure of the grown $TiO_2$ layers

The XRD pattern obtained from the titanium substrate after oxidation at 650 °C in air is given in Fig. S1. The pattern demonstrates peaks related to the metal substrate and the rutile layer grown; no other TiOx phase is detect. A comparison of counts recorded for different peaks clarifies that the polycrystalline rutile layer mainly consists of {110}-faceted crystallites.

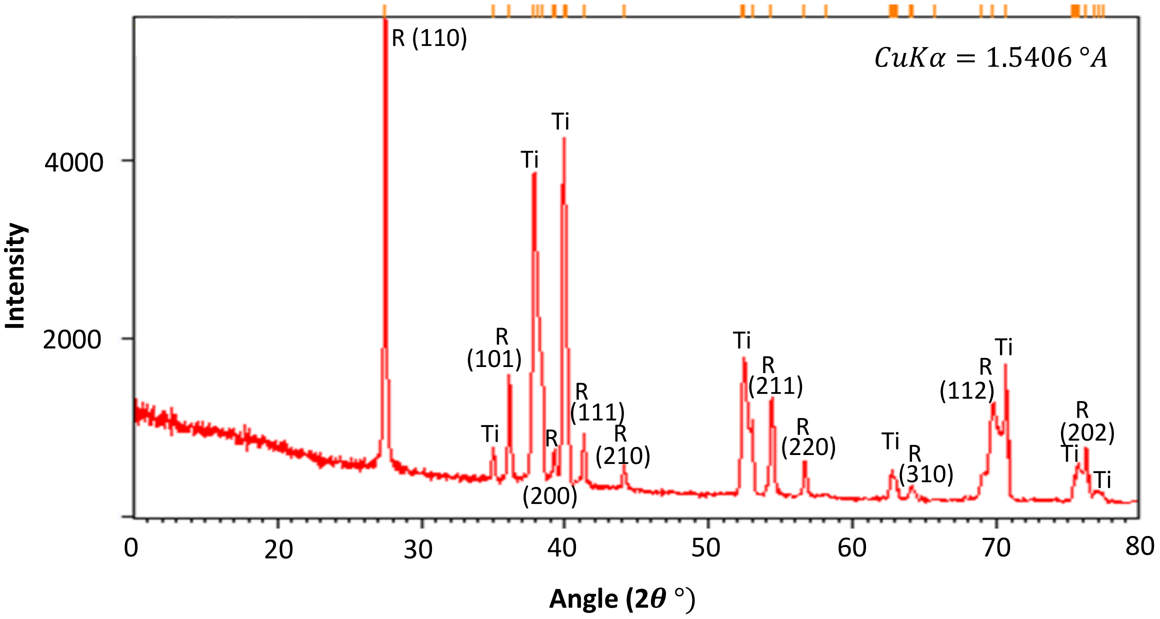


**Figure S1. The XRD pattern of the thermally grown $TiO_2$ on a Ti substrate at 650 °C;** the patter exhibits rutile and titanium peaks only.

## 2. Conduction monitoring during the filament formation and dissolution processes

Further experimental data is provided on the formation and dissolution of the conductive filaments in Ti/ poly-$TiO_2$/Ti structures.

After a long stay at zero bias conditions, a step voltage is applied to the Ti/poly-$TiO_2$/Ti samples. As shown in Fig. S2 a-d, an AC voltage waveform is added to the step biasing, which facilitates online monitoring of the device dynamic conductance ($G_d$) as well as the static conductance ($G_s$). According to Fig. S2 a and c, the A-sample operates almost like a normal resistor as its static and dynamic conductances remain nearly constant within the biasing field range examined.

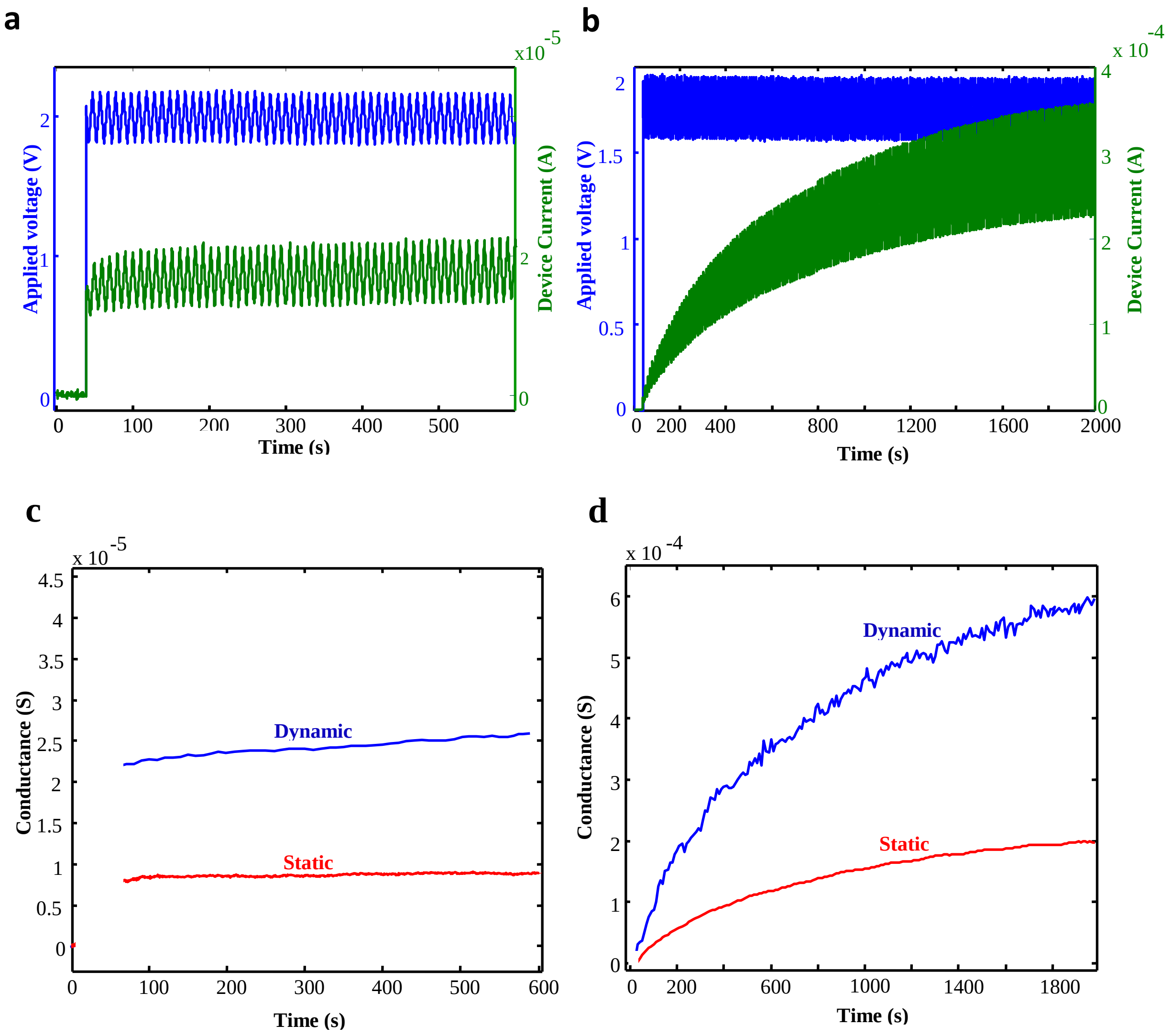


**Figure S2. The transient current and conductance variations in A- and B-samples in response to a step biasing voltage;** the applied voltage waveform and the device currents recorded in an A-sample (**a**) and a B-sample (**b**), and the temporal variations of the static and dynamic conductances of an A-sample (**c**) and a B-sample (**d**) after biasing field application.

However, Fig. S2b and d show that the B-sample performs differently as its dynamic conductance varies with time after biasing field application. This interesting feature of B-samples point to a number potential applications; e.g., the conductance of the device can be used as a measure of the time elapsed after the biasing voltage is switched on.

In another set of experiments, a constant DC biasing field, enough for filament formation, is applied to a B-sample. A small AC component added to the biasing field facilitates the online monitoring of the dynamic conductance of the device. Time is allowed for the device

current to reach its steady state level as shown in Fig. S2 b. Then, the temporal variations of the device current is recorded when the DC biasing field is suddenly dropped to different lower levels. The AC component of the biasing field is kept unchanged. The results are presented in Fig. S3 a-d. Based on these experimental results, the dynamic resistance of the device at any point in time is calculated and graphically presented in Fig. S4. The experimental results presented here are consistent with the filament formation and dissolution mechanism described in the manuscript.

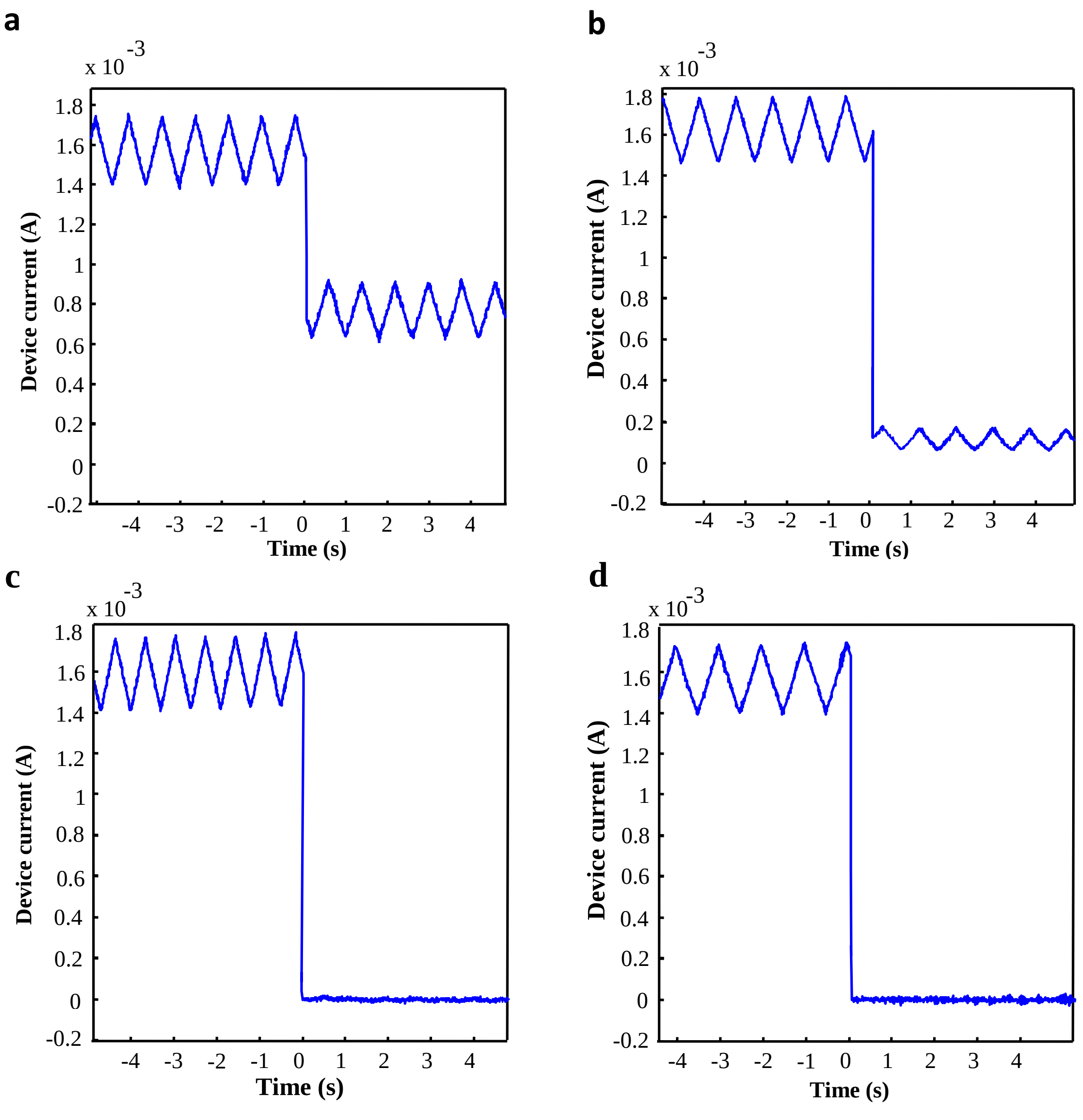


**Figure S3. The current fall in a B-sample due to a sudden drop in the DC biasing field** from 4 MV/m to (a) 3 MV/m, (b) 2 MV/m, (c) 1 MV/m, and (d) zero. The AC component of the biasing voltage is kept unchanged.

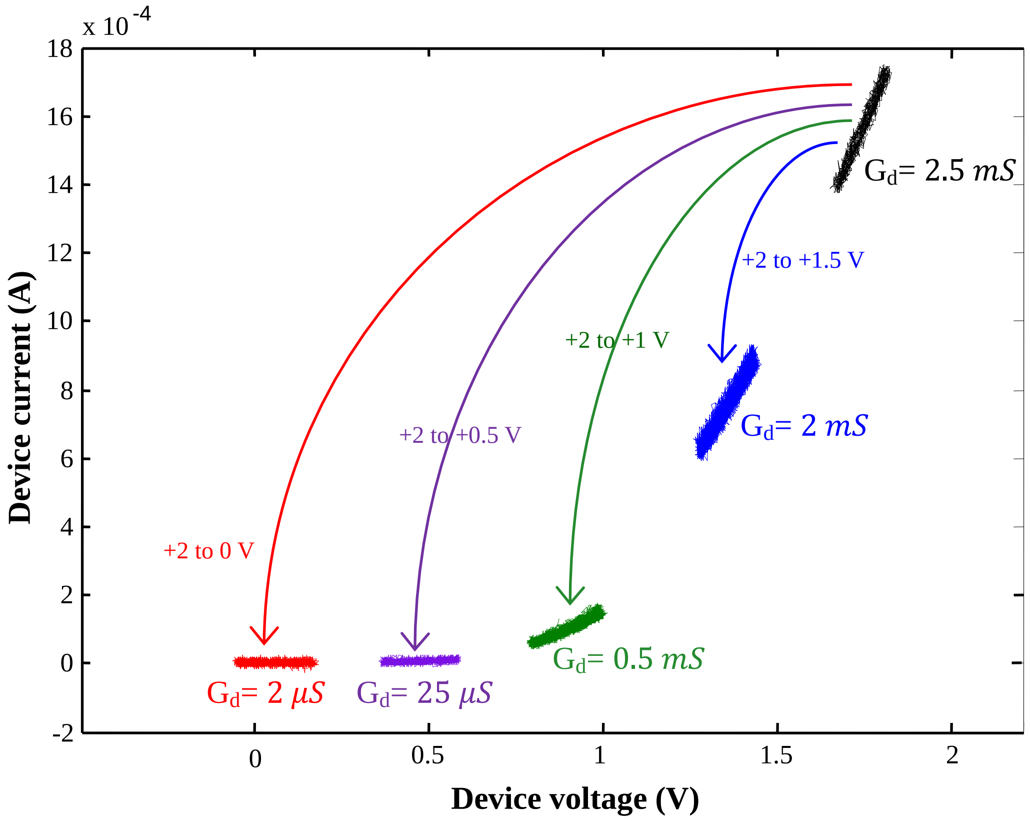


**Figure S4. Dropping of the dynamic conductance of a B-sample** due to the stated sudden biasing voltage variations.

**3. Is the observed asymmetric I-V diagram caused by a junction energy barrier?**

The I-V diagrams obtained for B-samples are vividly asymmetric and rectifying. The results of the following calculations rule out the possibility of this asymmetry being caused by a junction barrier:

Thermionic emission through a junction barrier results in a current given by [51]:

$$I_{TE} = I_0 \left( \exp\left(\frac{qV}{\eta KT}\right) - 1 \right) \quad \text{(S1)}$$

wherein $\eta$ is the ideality factor, q is the electronic charge, and $I_0$ is given by:

$$\mathrm{I_0 = AA^*T^2 \exp\left(-\frac{q\phi_b}{KT}\right)} \quad \text{(S2)}$$

in which $A$ is the junction area, $A^*$ is the Richardson constant, and $\emptyset_b$ is the energy barrier established at the junction. $\emptyset_b$ and $\eta$ follow from equations (S1) and (S2) :

$$\phi_b = \frac{KT}{q}\left(\frac{AA^*T^2}{I_0}\right) \tag{S3}$$

$$\eta = \frac{q}{KT}\left(\frac{d\,V}{d\,(lnI)}\right) \tag{S4}$$

$\emptyset_b$ and $\eta$ are calculated graphically from the semi-logarithmic I-V diagrams given as insets in Fig. 2c and d. The graphical work is presented in Fig. S5 a-b. The obtained $\emptyset_b$ and $\eta$ are given in Table-S1; $\eta$ values larger than 20 show that the observed I-V diagrams are distinct from those related to the thermionic emission over junction barriers.

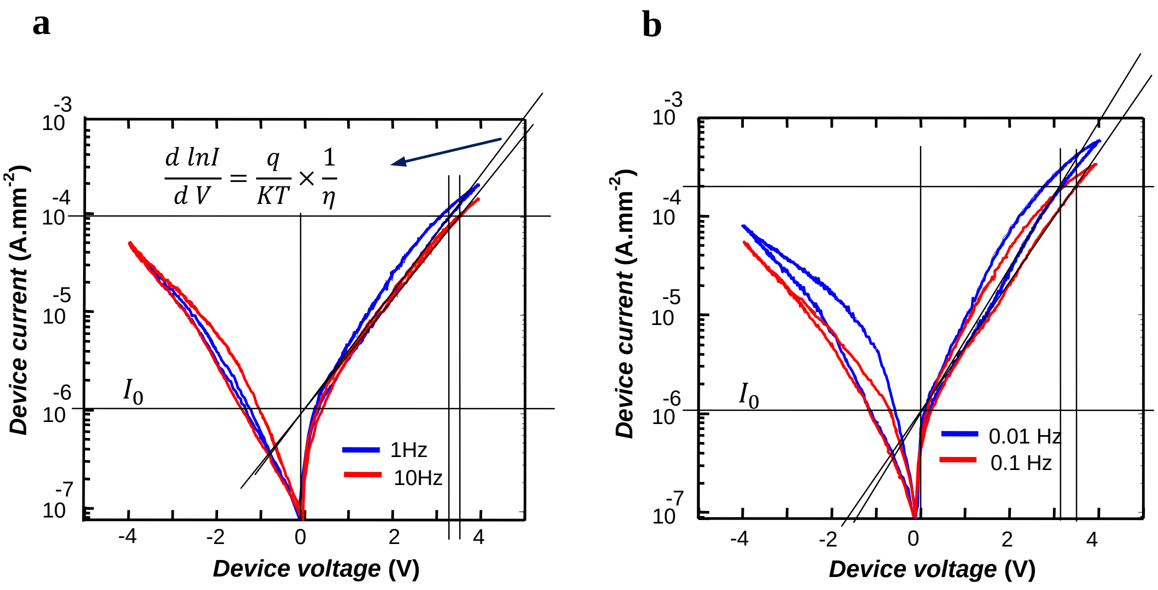


**Figure S5. Graphical calculations resulting $\emptyset_b$ and $\eta$ from the semi-logarithmic I-V diagrams**; the calculation results are given in Table S1.

**Table S1.** $\emptyset_b$ and $\eta$ values partly describing the IV diagrams given in Fig. S5.

| **Frequency** | $\eta$ | $\emptyset_b$(eV) |
|---|---|---|
| 10Hz | 29.7 | 0.72 |
| 1Hz | 27.5 | 0.72 |
| 0.1Hz | 25.6 | 0.72 |
| 0.01Hz | 23.4 | 0.72 |
